# 

**Published:** 1908-11-28

**Authors:** 


### GRADUATES' MEDICAL SCHOOLS.

DIARY FOR WEEK NOV. 30 to DEC. 4.

**THE ROYAL COLLEGE OF SURGEONS. Lincoln's Inn Fields, W.C.**

Bradshaw Lecture.

**At 5 p.m.**
**Dec. 4,** Sir W. Watson Cheyne, Bart., C.B.

**THE POST-GRADUATE COLLEGE, West London Hospital, Hammersmith, W.**

**At 10 a.m.**
**Nov. 30 and Dec. 3,** Mr. Etherington Smith, **Surgical Demonstration.**

**At 12 noon.**
**Nov. 30,** Dr. Low, **Pathological Demonstration.**

**At 12.15 p.m.**
**Dec. 4,** Dr. Pritchard, **Practical Medicine.**

**At 5 p.m.**
**Nov. 30,** Dr. Saunders, **Clinical, II.**
**Dec. 1,** Dr. Hood, **Some Clinical Observations upon Heart Disease, II.**
**Dec. 2,** Dr. Pritchard, **The Diagnosis and Treatment of Diphtheria.**
**Dec. 3,** Mr. Baldwin, **Practical Surgery, V.**
**Dec. 4,** Mr. Etherington Smith, **Fistula.**

**THE HOSPITAL FOR SICK CHILDREN, Great Ormond Street, W.C.**

**At 4 p.m.**
**Dec. 3,** Mr. Addison, **A Demonstration on Selected Surgical Cases.**

**LONDON SCHOOL OF CLINICAL MEDICINE, Seamen's Hospital, Greenwich, S.E.**

**At 2.15 p.m.**

**Dec. 1,** Professor Hewlett, **The Ætiology of Malignant Disease.**

**At 2.30 p.m.**
**Dec. 3,** Dr. Rankin, **Aphasia.**

**MEDICAL GRADUATES' COLLEGE AND POLYCLINIC, 22 Chenies Street, W.C.**

**At 5.15 p.m.**
**Nov. 30,** Dr. S. R. Wells, **Some Points in the Diagnosis and Treatment of Diseases of the Aortic Valve.**
**Dec. 1,** Dr. H. Campbell, **The Treatment of Heart Disease.**
**Dec. 2,** Dr. C. R. Box, **On Enlargements of the Epiphyses in Infants and Young Children.**
**Dec. 3,** Mr. Carless, **Some Points in the Surgery of the Biliary Passages.**

**MOUNT VERNON HOSPITAL, Hampstead.**

**At 5 p.m.**
**Dec. 3,** Dr. Kelynack, **Demonstration of Cases in the Wards.**

**ST. JOHN'S HOSPITAL FOR DISEASES OF THE SKIN, Leicester Square, W.C.**

**At 6 p.m.**
**Dec. 3,** Chesterfield Lectures, **Coccous Diseases.**

**NATIONAL HOSPITAL FOR THE PARALYSED AND EPILEPTIC, Queen Sq., Bloomsbury, W.C.**

**At 3.30 p.m.**

**Dec. 1 and 4,** Dr. Buzzard, **Myelitis.**

**THE THROAT HOSPITAL, Golden Square, W.**

**At 5.30 p.m.**
**Nov. 30,** Mr. Rose, **Tuberculosis and Syphilis of the Nose.**
**Dec. 3,** Mr. Parker, **Affections of the Septum and Alæ Nasi.**

**THE WEST-END HOSPITAL FOR NERVOUS DISEASES, Welbeck Street, W.**

**At 5 p.m.**
**Dec. 3,** Dr. Palmer, **Anterior Poliomyelitis.**

**HOSPITAL FOR CONSUMPTION AND DISEASES OF THE CHEST, Brompton, S.W.**

**At 4 p.m.**
**Dec. 2,** Dr. Horton-Smith Hartley, **Pneumothorax.**

THE HOSPITAL November 28, 1908.

*Name* ..................................................

*Address* ..................................................

..................................................

This Coupon must accompany manuscript or contributions intended for The Hospital.

